# Early-Life Cadmium Exposure and Child Development in 5-Year-Old Girls and Boys: A Cohort Study in Rural Bangladesh

**DOI:** 10.1289/ehp.1104431

**Published:** 2012-07-03

**Authors:** Maria Kippler, Fahmida Tofail, Jena D. Hamadani, Renee M. Gardner, Sally M. Grantham-McGregor, Matteo Bottai, Marie Vahter

**Affiliations:** 1 Institute of Environmental Medicine, Karolinska Institutet, Stockholm, Sweden; 2 International Centre for Diarrhoeal Disease Research, Bangladesh (icddr,b), Dhaka, Bangladesh; 3 Centre for International Health and Development, Institute of Child Health, University College London, London, United Kingdom

**Keywords:** cadmium exposure, child IQ, development, food pollutant, neurotoxicity, prenatal, urine

## Abstract

Background: Cadmium is a commonly occurring toxic food contaminant, but health consequences of early-life exposure are poorly understood.

Objectives: We evaluated the associations between cadmium exposure and neurobehavioral development in preschool children.

Methods: In our population-based mother–child cohort study in rural Bangladesh, we assessed cadmium exposure in 1,305 women in early pregnancy and their children at 5 years of age by measuring concentrations in urine (U-Cd), using inductively coupled plasma mass spectrometry. Children’s IQ at 5 years of age, including Verbal (VIQ), Performance (PIQ), and Full-Scale IQ (FSIQ), were measured by Wechsler Preschool and Primary Scale of Intelligence. Behavior was assessed by the Strengths and Difficulties Questionnaire (SDQ).

Results: In multiple linear regression models, adjusted for sex, home stimulation, socioeconomic status (SES), and maternal and child characteristics, a doubling of maternal U-Cd was inversely associated with VIQ (–0.84 points; 95% confidence interval: –1.3, –0.40), PIQ (–0.64 points; –1.1, –0.18), and FSIQ (–0.80 points; –1.2, –0.39). Concurrent child U-Cd showed somewhat weaker association with VIQ and FSIQ, but not PIQ. Stratification by sex and SES indicated slightly stronger associations with PIQ and FSIQ in girls than in boys and in higher-income compared with lower-income families. Concurrent U-Cd was inversely associated with SDQ-prosocial behavior and positively associated with SDQ-difficult behavior, but associations were close to the null after adjustment. Quantile regression analysis showed similar associations across the whole range of each developmental outcome.

Conclusion: Early-life low-level cadmium exposure was associated with lower child intelligence scores in our study cohort. Further research in this area is warranted.

There is a growing concern about the contribution of environmental chemicals to the increasing prevalence of developmental disabilities (Miodovnik 2011). The developing brain is particularly susceptible to toxic insult, as has been repeatedly demonstrated for toxic metals such as lead, mercury, manganese, and arsenic (e.g., Grandjean and Landrigan 2006). High doses of cadmium, a common contaminant in cereals and vegetables [European Food Safety Agency (EFSA) 2009], caused neurotoxicity in experimental animals (Petersson Grawe et al. 2004; Zhang et al. 2009), and some early studies reported associations of childhood cadmium exposure with mental retardation, learning difficulties, dyslexia, and deficits in visual motor tasks, IQ, and behavior in young children (Bonithon-Kopp et al. 1986; Jiang et al. 1990; Stellern et al. 1983; Thatcher et al. 1982). However, these were mostly case–control studies with few children and exposure assessment based on concentrations in hair, which may be subject to external contamination.

More recent studies are contradictory. A cross-sectional study of 7- to 16-year-old children in Chinese mining areas indicated associations between hair concentrations of cadmium, and particularly lead, and behavioral problems (Bao et al. 2009), but a small cross-sectional study in a U.S. mining area found no association between hair cadmium and neurophysiological function or behavior in children 11–13 years of age (Wright et al. 2006). In 5- to 7-year-old U.S. children participating in a lead-chelation trial, blood cadmium concentrations at 2 years of age were not associated with developmental measures (Cao et al. 2009). However, the lead exposure was extensive and the cadmium analysis had low sensitivity (40% of data below the detection limit). For children living close to a metal-smelter in China, high cord blood cadmium concentrations (median, 0.60 µg/L; *n* = 106) were negatively associated with children’s IQ at 4.5 years (Tian et al. 2009). Thus, firm evidence that cadmium affects child development is lacking.

The aim of the present study was to evaluate the impact of prenatal and concurrent cadmium exposure on children’s intelligence and behavior at 5 years of age in a large, population-based mother–child cohort.

## Materials and Methods

*Study Design.* This study on health effects of early-life exposure to environmental pollutants was nested into a food and micronutrient supplementation trial during pregnancy, in Matlab, Bangladesh (Tofail et al. 2008). In total, 4,436 women were recruited from November 2001 through October 2003, after detection of pregnancy by urine test. Participants were randomly assigned to one of three micronutrient supplementations, beginning in gestational week (GW) 14: 30 mg iron and 400 µg folic acid, 60 mg iron and 400 µg folic acid (standard program in Bangladesh), or multiple micronutrient supplement with 15 micronutrients, all in combination with either early (around GW9) or usual (GW16) food supplementation (Tofail et al. 2008).

Women with a positive pregnancy test (GW8, on average) were asked to donate a urine sample for assessment of environmental pollutants (initiated February 2002). All singleton babies born between May 2002 and December 2003 (*n* = 2,853) constituted the subcohort assessed for developmental measures (Tofail et al. 2008). For the present study, we selected the children born from August 2002 through September 2003 (*n* = 2,141) who had measures of cadmium in maternal (GW8) and child urine (5 years of age), data on basic demographic factors, maternal IQ, assessment of home environment, and developmental measures at 5 years of age (*n* = 1,305).

The study was approved by the relevant ethical review committees and conducted according to the principles of the Helsinki Declaration (World Medical Association 2008). Informed oral and written consent were obtained from the mothers, and all participants were free to refrain from any part of the study at any time.

*Exposure assessment.* Assessment of cadmium exposure was based on urinary cadmium (U-Cd) concentrations measured by inductively coupled plasma mass spectrometry (ICPMS; Agilent 7500ce, Agilent Technologies, Tokyo, Japan). The limits of detection were < 0.02 µg/L and < 0.002 µg/L when measuring cadmium in maternal urine and child urine, respectively, and no samples were below these limits. Details of sample collection, analytical method, and quality control have been described elsewhere (Kippler et al. 2009, 2010b, 2012b). Because both arsenic, commonly present in well water in the study area, and lead are known to affect child development (Grandjean and Landrigan 2006; Hamadani et al. 2011), we considered these exposures as well, using urinary concentrations of arsenic (U-As) and lead (U-Pb) (Bergkvist et al. 2010; Gardner et al. 2011). To compensate for variation in urine dilution, all measured concentrations were adjusted for specific gravity (Nermell et al. 2008).

*Outcome assessment.* We assessed children’s IQ at 5 years of age using the third edition of the Wechsler Preschool and Primary Scale of Intelligence (WPPSI) (Wechsler 2002). We used seven subtests of WPPSI. The sum of scaled scores (standardized by age) of verbal subtests (Information, 0–34 points; Vocabulary, 0–43 points; and Comprehension, 0–38 points), perfomance subtests (Block design, 0–40 points; Matrix reasoning, 0–29 points; and Picture completion, 0–32 points), formed the verbal IQ (VIQ) and performance IQ (PIQ), respectively, by adjusting to a mean of 100 (U.S. norms). The processing speed subtest (Coding, 0–65 points) is converted to scaled score. The sum of all seven scaled scores constituted the basis for Full Scale IQ (FSIQ).

To assess child behavior we applied, during a home visit, the Strengths and Difficulties Questionnaire (SDQ) (Goodman 2001), which consists of 25 questions to the parents concerning both positive and negative behavior of their child. There is one subscale for prosocial behavior (social strength; up to 10 points) and four subscales for difficult behaviors (emotional symptoms, conduct problems, hyperactivity/inattention, and peer relationship problems; up to 40 points in total). For SDQ-prosocial behavior, higher scores indicate more positive behavior, and for SDQ-difficult behavior higher scores indicate more problematic behavior.

We modified both the WPPSI and SDQ to make them culturally appropriate for Bangladeshi children, but without changing the underlying constructs. In piloting, both tests showed adequate test–retest reliability at 7-day intervals (intraclass correlation *r* > 0.90). We trained seven testers for conducting WPPSI and four interviewers for SDQ. To minimize tester-related bias the WPPSI testers were rotated across the four health clinics; to maintain quality we conducted regular supervision, and 5–10% of all tests were rated by a supervisor (interobserver reliability kappa > 0.92).

*Other measurements.* We used a modified version of Home Observation for Measurement of the Environment (HOME) (Caldwell 1967) to assess the amount of children’s stimulation at home. In addition, we assessed maternal IQ using Raven’s colored and progressive matrices (Raven et al. 2003).

Data on parental education, maternal weight, height, age, and birth order were recorded at enrollment. Socioeconomic status (SES) was defined through an asset index, constructed from extensive information on household ownership of different items (e.g., televisions and bicycles) and dwelling characteristics (e.g., drinking-water sources, house construction materials, and sanitation facilities). The assest index was generated through principal-component analysis and standardized as described previously (Gwatkin et al. 2000). In addition to birth anthropometry (Saha et al. 2008), body weight was measured by digital scale (TANITA HD–318; Tanita Corporation, Tokyo, Japan) and height by stadiometer (Seca 214, Leicester Height Measure; Seca GmbH & Co., Hamburg, Germany) at 5 years, and both were converted to age- and sex-standardized *z*-scores [weight-for-age (WAZ) and height-for-age (HAZ), using the World Health Organization growth references (de Onis et al. 2007)].

**Figure 1:**
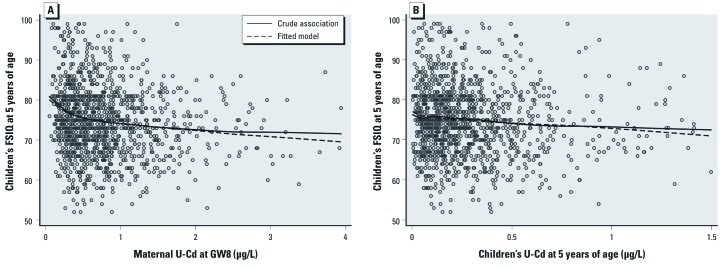
Associations of maternal U-Cd (*A*) and concurrent (children’s) U-Cd at 5 years of age (*B*) with FSIQ at 5 years of age. Solid lines represent Lowess-moving average curves; dashed lines represent fitted curves of FSIQ and U-Cd (log_2_-tansformed), adjusted for age at testing, tester, sex, birth order, birth weight, HAZ (5 years), HOME, maternal body mass index (early pregnancy), maternal IQ, SES, and maternal or concurrent urinary arsenic (log_2_-transformed). Twenty subjects are not included; 5 subjects with FSIQ < 50 points and 15 subjects with FSIQ > 100 points.

*Statistical analysis.* Data were analyzed using STATA (version 11; StataCorp, College Station, TX, USA). *p*-Values < 0.05 were considered statically significant. We first analyzed whether the developmental outcomes (VIQ, PIQ, FSIQ, SDQ-prosocial behavior, and SDQ-difficult behavior) at 5 years of age were associated with the two exposure measures (maternal U-Cd in early pregnancy and children’s concurrent U-Cd at 5 years, hereafter referred to as maternal and concurrent U-Cd, respectively) by examining scatterplots with a moving average Lowess curve (Figure 1). Because this examination indicated nonlinear relationships, exposure concentrations were log_2_-transformed in all further statistical analyses. We chose log_2_-transfomation because it provided a good fit of the data (Figure 1) and a simple interpretation of the beta-coefficient in the linear regression analysis (average change in outcome associated with doubling of the exposure).

We tested the crude associations between exposures, developmental outcomes, and potential confounders using Spearman correlation coefficient. We found strong correlations between SES and parental education, birth order and maternal age, weight, and head circumference at birth, and between children’s HAZ and WAZ (all *r*_s_ > 0.60; *p* < 0.001). To avoid multicolinearity education, maternal age, head circumference, and WAZ were not included in the further models. None of the mothers reported smoking during pregnancy, so smoking was not included in the models.

In model 1, using linear regression analysis, we estimated the association between each exposure and the different developmental outcomes at 5 years of age, adjusting only for child’s age at testing, tester, and sex. In model 2, we adjusted for previously reported predictors of the outcomes [child’s age at testing, tester, sex, birth order, birth weight, HAZ (5 years), HOME, maternal BMI (early pregnancy), maternal IQ, and SES]. In model 3, we entered both maternal and concurrent U-Cd into the same model, adjusting for the variables specified for model 2.

In sensitivity analyses, we tested if the associations between exposures and outcomes were affected by the food and micronutrient supplementation by additionally adjusting model 2 for either food (two groups) or micronutrient supplementation (three groups), or the combination of both (six groups).

We evaluated potential confounding by arsenic and lead exposure on the associations between cadmium and outcome by adding either maternal U-As or U-Pb (GW8; log_2_-transfomed), or children’s concurrent U-As or U-Pb (log_2_-transfomed) to the variables defined in model 2. Finally, we considered all three exposures in the same model, one for maternal and one for concurrent exposure. Because U-Pb was available for only 779 mothers, was not significantly associated with any of the outcomes, and did not markedly influence the association with cadmium, it was not included in the further analyses.

For estimation of effect size, we used the model estimates from model 2 to estimate the difference in outcomes between children with cadmium exposure ≥ 95th percentile compared with children ≤ 5th percentile using Stata’s lincom command.

We assessed the possibility that cadmium exposure may affect boys and girls differently by stratifying model 2 (including adjustment for arsenic exposure) for sex, and by evaluating multiplicative interactions between sex (boy = 0, girl = 1) and the different exposures (continuous log_2_-transformed). We repeated the same procedure stratifying for SES (median split; low = 0, high = 1).

Finally, we examined whether the associations between exposure and outcome varied across the distribution of developmental outcomes using multivariable-adjusted quantile regression analyses, adjusted as model 2 with U-As. We estimated changes in the 25th, 50th, and the 75th percentiles of the different developmental outcomes associated with a doubling of maternal or concurrent U-Cd modeled as a log_2_-transformed variable. The estimates, confidence intervals, and *p*-values were based on 200 bootstrap samples. Wald test was used to test for difference between different quantiles.

## Results

Of the initial 2,141 children born within the specified period, 836 (39%) had incomplete information; 446 had no developmental measures at 5 years, 284 had no maternal urine sample, 35 had no concurrent urine sample, and 71 had no measures of home stimulation. Reasons for missing developmental measures were refusal (*n* = 46), not available on several visits (*n* = 382), migration (*n* = 8), death (*n* = 6), disability (*n* = 1), and illness (*n* = 3). Children who were not tested came from families with slightly higher SES, education, and maternal BMI, and lower birth order, but there was no significant difference in maternal U-Cd between tested and nontested children (data not shown).

The 1,305 children included in the study were on average 5.3 years old at the time of testing (Table 1), with 41% being underweight (WAZ < –2SD) and 33% stunted (HAZ < –2SD).

**Table 1:** Characteristics of the families and their children at birth and 5 years of age.

Variable	All children (*n* = 1,305)	Boys (*n* = 668)	Girls (*n* = 637)
Family characteristics
Maternal age (years)	26 ± 5.9	26 ± 5.8	26 ± 6.1
Maternal BMI (kg/m2)a	20 ± 2.5	20 ± 2.6	20 ± 2.5
Maternal educationb	4.7 ± 4.0	4.6 ± 4.0	4.8 ± 4.1
Paternal educationb	5.1 ± 4.6	5.0 ± 4.5	5.2 ± 4.7
SESc	–0.19 ± 2.4	–0.23 ± 2.3	–0.15 ± 2.4
Maternal hemoglobin (g/dL)d	11.5 (9.8–13.9)	11.6 (9.8–13.7)	11.6 (9.8–14.0)
Maternal U-Cd (µg/L)e	0.63 (0.18–2.0)	0.62 (0.18–1.9)	0.63 (0.19–2.1)
Maternal U-As (µg/L)e	86 (18–524)	84 (17–501)	90 (18–546)
Maternal U-Pb (µg/L)e,f	2.8 (1.1–6.8)	2.8 (1.2–7.2)	2.7 (1.1–6.3)
Children’s characteristics at birth
Birth order	1.4 ± 1.4	1.4 ± 1.4	1.4 ± 1.4
Birth weight (g)	2,680 ± 392	2,716 ± 411	2,642 ± 367
Birth length (cm)	48 ± 2.3	48 ± 2.4	48 ± 2.1
Head circumference (cm)	32 ± 1.7	33 ± 1.8	32 ± 1.6
Children’s characteristics at 5 years of age
Age at testing (years)	5.3 ± 0.18	5.3 ± 0.20	5.3 ± 0.16
HAZ	–1.6 ± 0.95	–1.6 ± 1.0	–1.6 ± 0.90
WAZ	–1.8 ± 0.90	–1.7 ± 0.93	–1.9 ± 0.87
U-Cd (µg/L)g	0.22 (0.078–0.63)	0.22 (0.073–0.59)	0.23 (0.080–0.69)
U-As (µg/L)g	53 (17–364)	52 (17–381)	54 (17–334)
U-Pb (µg/L)g	3.8 (1.6–11)	3.8 (1.6–9.7)	3.8 (1.6–11)
Verbal raw score	19 ± 5	19 ± 5	19 ± 6
Performance raw score	19 ± 5	19 ± 5	19 ± 5
Processing speed raw score	13 ± 9	12 ± 9	13 ± 9
VIQ	80 ± 9.6	79 ± 9.3	80 ± 9.8
PIQ	76 ± 9.9	76 ± 9.8	76 ± 9.9
FSIQ	75 ± 9.6	75 ± 9.4	75 ± 9.8
SDQpro	6.7 ± 1.9	6.4 ± 1.9	7.0 ± 1.8
SDQdiff	13 ± 3.9	13 ± 3.8	12 ± 3.9
Biomarkers are presented as median (5th–95th percentiles); other variables are given as mean ± SD. aGestational week 8. bYears of formal schooling. cStandardized with a mean of zero. dGW14. eGW8, adjusted for specific gravity 1.012 g/mL. fn = 779. gAt 5 years of age, adjusted for specific gravity 1.012 g/mL.						

In bivariate analyses, especially maternal but also concurrent U-Cd were correlated with developmental outcomes (Table 2). In the linear regression analyses (Table 3), both maternal and concurrent U-Cd concentrations were inversely associated with VIQ [β = –1.5; 95% confidence interval (CI): –2.0, –1.1, and β = –1.2; 95% CI: –1.8, –0.72, respectively], PIQ (β = –1.4; 95% CI: –1.9, –0.87, and β = –1.4; 95% CI: –1.9, –0.80, respectively), and FSIQ (β = –1.6; 95% CI: –2.1, –1.1, and β = –1.4; 95% CI: –2.0, –0.90, respectively). In general associations with these outcomes, especially for maternal U-Cd (VIQ β = –0.84; 95% CI: –1.3, –0.40; PIQ β = –0.64; 95% CI: –1.1, –0.18; and FSIQ β = –0.80; 95% CI: –1.2, –0.39), remained after further adjustment. For the behavioral measures (Table 3), concurrent U-Cd was inversely associated with SDQ-prosocial behavior and positively associated with SDQ-difficult behavior, but these associations were close to the null after adjustment.

**Table 2:** Spearman’s rank correlation coefficients (*p*-value) of associations between maternal U-Cd (GW8), concurrent U-Cd (5 years of age), and child developmental measures at 5 years of age (*n* = 1,305).

Variables	Maternal U-Cd^a^	Concurrent U-Cd^b^
VIQ	–0.16 (< 0.001)	–0.12 (< 0.001)
PIQ	–0.14 (< 0.001)	–0.12 (< 0.001)
FSIQ	–0.16 (< 0.001)	–0.13 (< 0.001)
SDQpro	–0.027 (0.33)	–0.057 (0.038)
SDQdiff	0.041 (0.13)	0.046 (0.099)
Maternal U-Cd ( µg/L)a	—	0.098 (< 0.001)
Concurrent U-Cd ( µg/L)b	0.098 (< 0.001)	—
aGW8, adjusted to specific gravity 1.012 g/mL. bAt 5 years of age, adjusted to specific gravity 1.012 g/mL.				

**Table 3:** Multivariable-adjusted associations of maternal U-Cd and concurrent U-Cd (both log2-transfomed) with child developmental measures at 5 years of age (n = 1,305).

Predicted	Maternal U-Cd			Concurrent U-Cd		
	β (95% CI)^a^	*p*-Value	β (95% CI)^a^	*p*-Value
VIQ
Model 1	–1.5 (–2.0, –1.1)	< 0.001	–1.2 (–1.8, –0.72)	< 0.001
Model 2	–0.84 (–1.3, –0.40)	0.001	–0.45 (–0.93, 0.039)	0.072
Model 3	–0.81 (–1.3, –0.38)	< 0.001	–0.37 (–0.85, 0.11)	0.14
PIQ
Model 1	–1.4 (–1.9, –0.87)	< 0.001	–1.4 (–1.9, –0.80)	< 0.001
Model 2	–0.64 (–1.1, –0.18)	0.007	–0.69 (–1.2, –0.18)	0.008
Model 3	–0.59 (–1.1, –0.13)	0.013	–0.64 (–1.2, –0.13)	0.015
FSIQ
Model 1	–1.6 (–2.1, –1.1)	< 0.001	–1.4 (–2.0, –0.90)	< 0.001
Model 2	–0.80 (–1.2, –0.39)	< 0.001	–0.62 (–1.1, –0.16)	0.008
Model 3	–0.76 (–1.2, –0.34)	< 0.001	–0.55 (–1.0, –0.088)	0.020
SDQpro
Model 1	–0.050 (–0.15, 0.046)	0.30	–0.12 (–0.23, –0.014)	0.027
Model 2	0.0072 (–0.090, 0.10)	0.88	–0.061 (–0.17, 0.046)	0.26
Model 3	0.012 (–0.085, 0.11)	0.81	–0.062 (–0.17, 0.045)	0.26
SDQdiff
Model 1	0.13 (–0.066, 0.33)	0.19	0.29 (0.073, 0.52)	0.009
Model 2	–0.0024 (–0.20, 0.19)	0.98	0.11 (–0.11, 0.33)	0.31
Model 3	–0.011 (–0.21, 0.19)	0.91	0.11 (–0.11, 0.34)	0.31
Model 1: Maternal and child exposure evaluated separately, adjusted for age at testing, tester, and sex. Model 2: Maternal and child exposure evaluated separately, adjusted for age at testing, tester, sex, birth order, birth weight, HAZ (5 years), HOME, maternal body mass index (early pregnancy), maternal IQ, and SES. Model 3: Evaluation of joint maternal and concurrent urinary cadmium, adjusted for age at testing, tester, sex, birth order, birth weight, HAZ (5 years), HOME, maternal body mass index (early pregnancy), maternal IQ, and SES. aEstimated change in the outcome score with a doubling of U-Cd.								

In sensitivity analyses, we additionally adjusted model 2 for food and micronutrient supplementations, but none of those markedly changed (< 2%) any of the effect estimates for cadmium on children’s intelligence or behavior (data not shown). Additional adjustment for maternal or concurrent U-As only slightly decreased the U-Cd effect estimates for VIQ and FSIQ, but adjusting for U-As had little or no influence on associations between U-Cd and PIQ [see Supplemental Material, Table S1 (http://dx.doi.org/10.1289/ehp.1104431)]. Maternal and concurrent U-Pb were not significantly associated with any of the outcomes, and adjustment for U-Pb had little influence on associations between U-Cd and children’s PIQ, although associations with VIQ and FSIQ increased somewhat. Finally, we combined all three exposures into the same model, with similar results (see Supplemental Material, Table S1).

**Figure 2:**
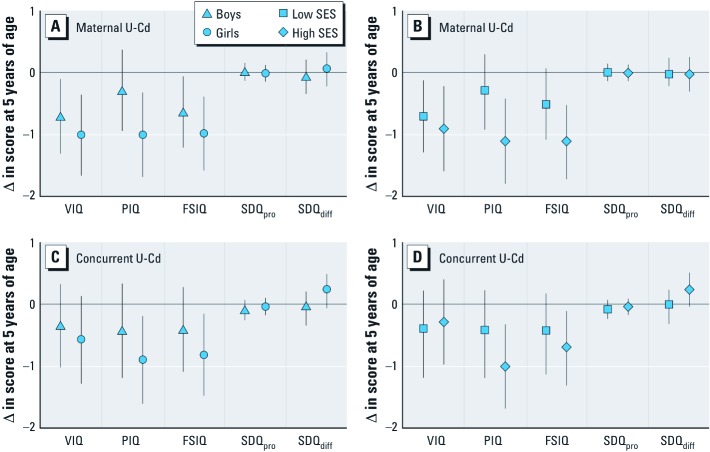
Multivariable-adjusted associations between (*A*,*B*) maternal U-Cd (log_2_-transformed) and (*C*,*D*) concurrent U-Cd (log_2_-transformed) with developmental outcomes at 5 years of age after stratification by sex (*A*,*C*) or SES (*B*,*D*). Estimates with 95% CIs represent the change in outcome score with a doubling of U-Cd exposure adjusted for age at testing, tester, birth order, birth weight, HAZ (5 years), HOME, maternal body mass index (early pregnancy), maternal IQ, maternal or concurrent U-As (log_2_-transformed).

Independent sample *t*-tests showed higher concurrent U-Cd concentrations (*p* = 0.013), more SDQ-difficult behavior (*p* < 0.001), and less SDQ-prosocial behavior in boys than in girls. In the multivariable-adjusted analyses stratified by sex (Figure 2A,C), maternal U-Cd was inversely associated with VIQ, PIQ, and FSIQ in both boys and girls. In general, the associations were slightly more pronounced in girls, and similar patterns were observed for associations with concurrent U-Cd. We formally tested for a potential interaction between log_2_-transfomed cadmium and sex; *p*-values were 0.13 and 0.23 for PIQ with maternal and concurrent U-Cd, respectively, and 0.31–0.95 in all other models. Stratification by SES (median split; Figure 2B,D) showed inverse associations between both maternal and concurrent U-Cd and VIQ, PIQ and FSIQ in families with both low and high SES. Except for the association between concurrent U-Cd and VIQ, there was a tendency of more pronounced associations in families with high SES compared with those with lower SES. *p*-Values for interaction were 0.085 and 0.31 for PIQ with maternal and concurrent U-Cd, respectively, and 0.40–0.95 in all other models.

Multivariable-adjusted quantile regression analyses [see Supplemental Material, Figure S1 (http://dx.doi.org/10.1289/ehp.1104431)] showed that estimated changes in the values of the 25th, 50th, and 75th percentiles of VIQ, PIQ, and FSIQ associated with a doubling of maternal and concurrent U-Cd were fairly similar across the entire distribution of IQ. Concurrent U-Cd was associated with lower values for the median and 75th percentile of SDQ-prosocial behavior scores, but none of the associations were significant. We found no significant differences among the quantile-specific effect estimates for any outcome.

**Figure 3:**
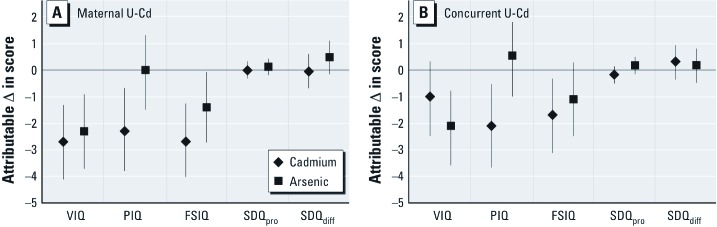
Multivariable-adjusted cadmium-related differences in child development (with 95% CIs), calculated by comparing the model predicted developmental measures of children with maternal (*A*) and concurrent (*B*) U-Cd and U-As ≥ 95th percentile (maternal U-Cd 2 µg/L and U-As 524 µg/L; concurrent U-Cd 0.63 µg/L and U-As 364 µg/L) to those ≤ 5th percentile (maternal U-Cd 0.18 µg/L and U-As 18 µg/L; concurrent U-Cd 0.078 µg/L and U-As 17 µg/L). Estimates with 95% CIs are adjusted for, beside cadmium and arsenic, age at testing, tester, sex, birth order, birth weight, HAZ (5 years), HOME, maternal body mass index (early pregnancy), maternal IQ, and SES.

In the effect size calculation, maternal U-Cd ≥ 95th percentile was associated with a multivariable-adjusted decrement of about 2.7 points in FSIQ (95% CI: –4.2, –1.2) compared with the maternal U-Cd ≤ 5th percentile, and similar decreases were seen in VIQ and PIQ (Figure 3). Compared with concurrent U-Cd ≤ 5th percentile, concurrent U-Cd ≥ 95th percentile was associated with a decrement of 1.7 points in FSIQ (95% CI: –3.1, –2.5) and 2.1 points in PIQ (95% CI: –3.7, –0.55), whereas the decrease in VIQ was nonsignificant (–1 point; 95% CI: –2.5, 0.47). Estimates of associations for high compared with low U-As from the same models indicated that high prenatal arsenic exposure (≥ 95th percentile for maternal U-As) was associated with a 1.4-point decrement in FSIQ (95% CI: –2.7, –0.03) and a 2.3-point decrement in VIQ (95% CI: –3.7, –0.86), whereas the change in PIQ was nonsignificant (0.12; 95% CI: –0.19, 0.44) (Figure 3). High concurrent arsenic exposure was associated with a decrement of 2.1 points in VIQ (95% CI: –3.6, –0.62), and nonsignificant changes in FSIQ (–1.1; 95% CI: –2.5, 0.28) and PIQ (0.55; 95% CI: –1.0, 2.1).

## Discussion

Our findings provide new evidence of an association between exposure to cadmium at low levels and lower IQ scores in children. The associations between maternal U-Cd during pregnancy and children’s VIQ, PIQ, and FSIQ at 5 years of age seemed robust and persisted after controlling for multiple confounders, including the quality of home stimulation, maternal IQ, birth order, and SES. Childhood cadmium exposure seemed somewhat less influential than the maternal exposure during pregnancy for VIQ, but not for PIQ. Further, the inverse associations of cadmium with child IQ, especially PIQ, seemed to be slightly more pronounced in girls than in boys, in families with higher than lower SES, and was about the same at low, median, and high IQ levels. The highest (≥ 95th percentile) maternal and concurrent child exposures (≥ 2 and ≥ 0.6 µg/L in urine, respectively) were associated with 2.7- and 1.7-point reductions in FSIQ points, respectively, compared with the lowest exposure levels (≤ 0.18 and ≤ 0.08 µg/L, respectively). In line with the present finding, we recently found an inverse association between maternal cadmium exposure in the present cohort and size of daughters at birth, particularly the head circumference (Kippler et al. 2012b), which may influence childhood IQ (Gale et al. 2006). There was little evidence for any association in the sons.

The diet was probably the main source of cadmium exposure, as none of the women smoked and they lived in a rural environment with essentially no industrial contamination. The rice-based diet in this population contributes 20–35 µg cadmium daily (Kippler et al. 2010b, 2012b). This is somewhat higher dietary cadmium exposure than in Western countries with typically more mixed diets, but similar to vegetarian or rice-based diets in other countries (EFSA 2009; Ikeda et al. 2011). Similarly, the maternal U-Cd concentrations (median, 0.63 µg/L) were slightly higher than in women in Western countries such as the United States (median, ~ 0.21 µg/L) and Sweden (median, 0.31 µg/L) [Åkesson et al. 2002; Centers for Disease Control and Prevention (CDC) 2010].

The main strengths of this study include the large sample size and wide range of environmental cadmium exposure, the prospective population-based design, and evaluation of multiple potential confounders. We measured cadmium concentrations in urine, the commonly used biomarker of long-term cadmium exposure (half-life, 10–30 years) (Järup and Åkesson 2009), using a sensitive and reliable ICPMS method. Cord blood would probably be the optimal biomarker for assessing the actual prenatal cadmium exposure, as the placenta constitutes a partial barrier against fetal cadmium exposure (Kippler et al. 2010a). However, we previously showed that maternal U-Cd was associated with both Cd in cord blood, with Cd accumulation in placenta, and with infant Cd exposure in the study cohort (Kippler et al. 2010a, 2010b), suggesting that all of these exposure biomarkers may be associated with child development.

A limitation of our study is the loss to follow-up for testing at 5 years of age (34%). However, differences between tested and nontested children were minor. The WPPSI scores have not been standardized for Bangladesh compared with Western countries, but the relatively low overall IQ scores are consistent with other studies in low-income populations, and likely related to cultural and socioeconomic differences compared with Western countries (Riojas-Rodriguez et al. 2010; Wasserman et al. 2007). The test results showed good test–retest and interobserver reliability, and they correlated with SES, education, maternal age, BMI, and IQ, and children’s anthropometry in theoretically expected ways. Another limitation is that we assessed child nutrition only via anthropometric measures because no nutritional biomarkers were available, nor did we have information about childhood illnesses or secondhand smoking.

Besides being moderately exposed to cadmium, many of the women and children had high arsenic exposure through drinking water, which we previously found associated with imparied child VIQ (Hamadani et al. 2011). Adjusting for maternal and concurrent U-As slightly decreased the associations between cadmium and child VIQ, but it did not affect PIQ. Unexpectedly, the highest level of maternal cadmium exposure in the study population, which is still moderate compared with exposures in other populations, was more strongly associated with child IQ than the highest level of arsenic exposure (> 500 µg/L), which is much greater than the typical background concentrations of < 10 µg/L (CDC 2010). An exception was the association between concurrent U-As exposure and VIQ, which was stronger than the association between concurrent U-Cd and VIQ.

Similarly, adjustment for maternal and childhood exposure to lead did not markedly change the associations between cadmium and child IQ. The found U-Pb concentrations indicate higher exposure in the study population (median, 2.8 and 3.8 µg/L for maternal and concurrent levels, respectively) than in U.S. women and children (median, ~ 0.7 µg/L), and more similar to that observed in Japanese women (median, 1.2 µg/L) (CDC 2010; Fukui et al. 2004). Still, we found no significant association of U-Pb with children’s IQ or behavior. U-Pb has been proposed as an alternative to blood lead as an exposure biomarker, because it is related to plasma lead (Bergdahl et al. 1997), which is the biologically active fraction. However, U-Pb is likely to have a larger day-to-day variation than blood-lead, and we only had maternal U-Pb concentrations in early pregnancy for a subsample of the mothers. Also, the exposure at this point in time may not adequately reflect the exposure during later fetal development and during breast-feeding, because blood lead concentrations increased markedly during pregnancy and lactation, particularly in undernourished mothers (Bergkvist et al. 2010). Thus, further studies are needed to clarify potential associations between lead exposure and child development in this population.

There are at least three possible mechanisms by which cadmium may directly or indirectly affect brain development. A direct effect on neuronal cells is plausible because cadmium is a potent pro-oxidant (Mates et al. 2010) and, possibly, even the limited amounts transported to the fetus (Kippler et al. 2010a) may cause oxidative stress in the sensitive developing brain. Indeed, we found associations between cadmium exposure and oxidative stress in both the studied mothers (Engström et al. 2010) and their infants (Kippler et al. 2012a). In addition, dopaminergic markers were associated with cadmium exposure in a cross-sectional study of European children (de Burbure et al. 2006), and experimental studies have shown that cadmium may interfere with neuronal differentiation (Gulisano et al. 2009) and neurotransmitters (Andersson et al. 1997).

Second, the marked accumulation of cadmium in placenta may lead to decreased zinc transport to the fetus (Kippler et al. 2010a). Zinc is important for brain development (Bhatnagar and Taneja 2001), and deficiency during the rapid period of brain growth may have long-term consequences for child development (Salgueiro et al. 2002). A third possible mode of action of cadmium is hormonal interactions, particularly with estrogen (Johnson et al. 2003), thyroid hormones (Iijima et al. 2007; Ishitobi et al. 2007), and growth hormones (Turgut et al. 2005), all of which are important for brain development. We observed some differences in associations by sex that may be consistent with hormonal interactions. Interestingly, stronger associations with IQ scores in girls compared with boys were recently reported for arsenic (Hamadani et al. 2011) and manganese (Bouchard et al. 2011; Riojas-Rodriguez et al. 2010). However, in the present study the contribution of social factors to differences in estimated effects, such as sex differences in nutritional status, home stimulation, and care-seeking behaivor, cannot be excluded given the male child preference in rural Bangladesh (Khatun et al. 2004).

## Conclusions

Our findings suggest that early-life cadmium exposure, at levels present in most countries, may be harmful for brain development. The results need verification in other populations. In particular, the evidence of possible sex or sex-related associations are intriguing and provide suggestions for future mechanistic studies.

## Supplemental Material

(397 KB) PDF
