# Supplementary material for: Early-Life Cadmium Exposure and Child Development in 5-Year-Old Girls and Boys: A Cohort Study in Rural Bangladesh
Source: Environ Health Perspect. 2012 Jul 3;120(10):1462–8. doi: 10.1289/ehp.1104431 (PMC3491924; doi:10.1289/ehp.1104431)
Supplement: (397 KB) PDF [file ehp.1104431.s001.pdf]

Supplemental Material

Early-Life Cadmium Exposure and Child Development in 5-Year-Old Girls and Boys: a Cohort Study in Rural Bangladesh

Maria Kippler[1#], Fahmida Tofail[1,2#], Jena D Hamadani[2], Renee M Gardner[1], Sally M Grantham-McGregor[3], Matteo Bottai[1], Marie Vahter[1]*

[1]Institute of Environmental Medicine, Karolinska Institutet, Stockholm, Sweden; [2]International Centre for Diarrhoeal Disease Research, Bangladesh (ICDDR,B), Dhaka, Bangladesh; [3]Centre for International Health and Development, Institute of Child Health, University College London, London, UK

[#]Authors contributed equally to the manuscript.

*Marie Vahter, Institute of Environmental Medicine, Division of Metals and Health, Karolinska Institutet, Box 210, SE-171 77, Stockholm, Sweden. Telephone: +46 8 524 875 40. Telefax: +46 8 33 69 81. Email: marie.vahter@ki.se

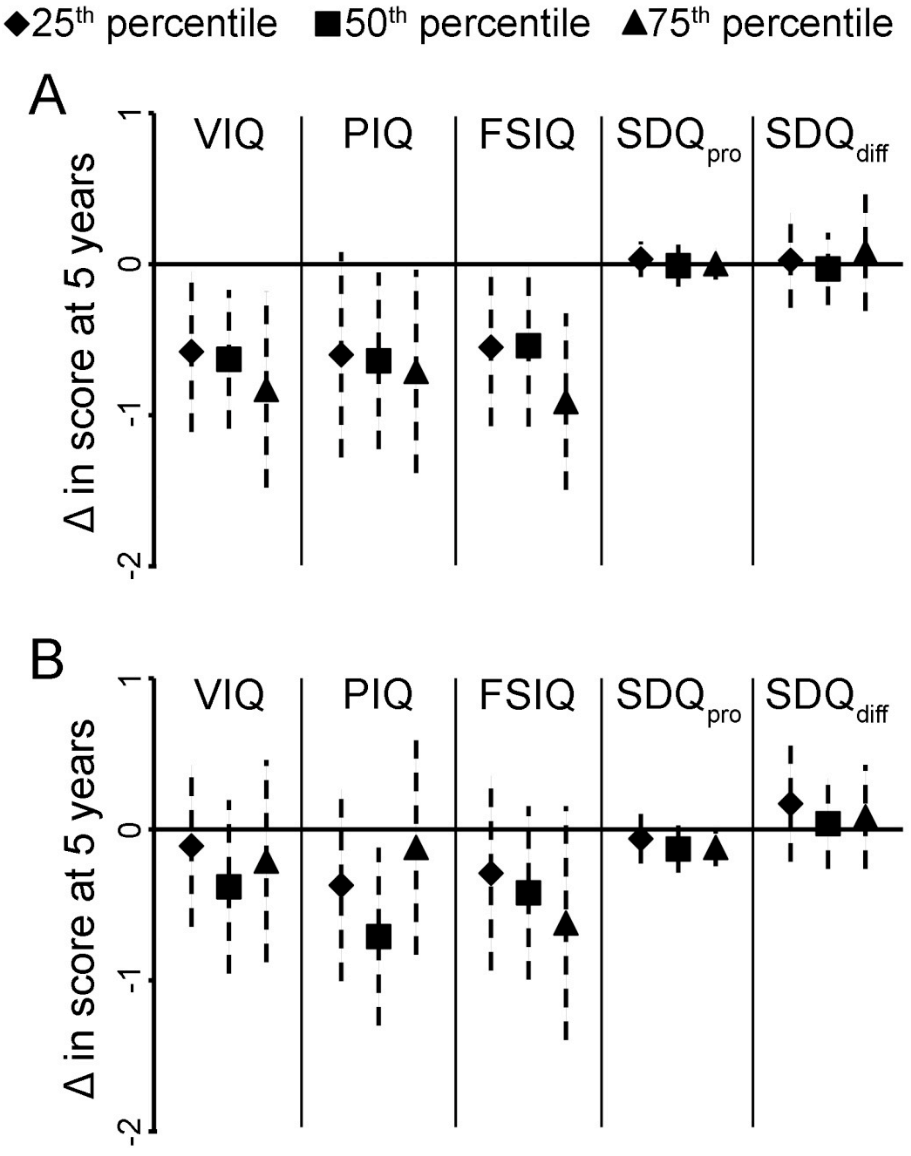

Supplemental Material, Figure S1 Multivariable-adjusted quantile regression analysis of associations between **A**) maternal urinary cadmium (U-Cd; early pregnancy; log_2-transformed) and **B**) concurrent childhood U-Cd (log_2-transformed) with children's developmental measures (25th percentile: diamonds, 50th percentile: squares, and 75th percentile: triangles) at 5 years of age. Estimates with 95% CI represent the change in the values of the 25th, 50th and 75th percentiles of the outcome scores with a doubling of U-Cd exposure adjusted for age at testing, tester, sex, birth order, birth weight, HAZ (5 years), HOME, maternal BMI (early pregnancy), maternal IQ, SES, and maternal or concurrent urinary arsenic (log_2-transformed).

Supplemental Material, Table S1 Multivariable-adjusted linear regression analyses of cadmium exposure (maternal or concurrent urinary cadmium; $\log_2$-transformed) and developmental outcomes at 5 years of age after additionally adjusting for arsenic or/and lead exposure (maternal or concurrent urine concentrations; $\log_2$-transformed).

	**VIQ**	**PIQ**	**FSIQ**	**SDQ_{pro}**	**SDQ_{diff}**
Predictors	β (95% CI; *p*)	β (95% CI; *p*)	β (95% CI; *p*)	β (95% CI; *p*)	β (95% CI; *p*)
**Model A**[a]					
Maternal Cd	-0.78 (-1.2, -0.34; *0.001*)	-0.65 (-1.1, -0.18; *0.006*)	-0.77 (-1.2, -0.35; *<0.001*)	0.00016 (-0.098, 0.098; *0.99*)	-0.015 (-0.22, 0.19; *0.88*)
Maternal As	-0.47 (-0.76, -0.18; *0.002*)	0.0013 (-0.31, 0.31; *0.99*)	-0.28 (-0.56, -0.0069; *0.045*)	0.026 (-0.039, 0.090; *0.44*)	0.098 (-0.035, 0.23; *0.15*)
**Model B**[a]					
Concurrent Cd	-0.33 (-0.82, 0.16; *0.18*)	-0.70 (-1.2, -0.18; *0.008*)	-0.55 (-1.0, -0.078; *0.022*)	-0.059 (-0.17, 0.049; *0.28*)	0.11 (-0.12, 0.33; *0.36*)
Concurrent As	-0.47 (-0.81, -0.14; *0.005*)	0.12 (-0.23, 0.48; *0.49*)	-0.25 (-0.57, 0.063; *0.12*)	0.039 (-0.035, 0.11; *0.30*)	0.042 (-0.11, 0.19; *0.59*)
**Model C**[a]					
Maternal Cd	-1.2 (-1.8, -0.58; *<0.001*)	-0.63 (-1.3, 0.015; *0.055*)	-0.98 (-1.6, -0.37; *0.001*)	0.033 (-0.11, 0.17; *0.65*)	0.071 (-0.22, 0.36; *0.63*)
Maternal Pb	0.53 (-0.23, 1.3; *0.17*)	-0.19 (-0.96, 0.57; *0.62*)	0.10 (-0.60, 0.81; *0.77*)	0.052 (-0.11, 0.21; *0.54*)	0.29 (-0.053, 0.63; *0.098*)

Supplemental Material, Table S1 (continued)

Predictors	**VIQ** β (95% CI; *p*)	**PIQ** β (95% CI; *p*)	**FSIQ** β (95% CI; *p*)	**SDQ_{pro}** β (95% CI; *p*)	**SDQ_{diff}** β (95% CI; *p*)
**Model D**[a]					
Concurrent Cd	-0.51 (-1.0, -0.015; *0.044*)	-0.73 (-1.2, -0.21; *0.006*)	-0.68 (-1.1, -0.22; *0.004*)	-0.057 (-0.17, 0.051; *0.30*)	0.12 (-0.10, 0.35; *0.29*)
Concurrent Pb	0.38 (-0.16, 0.92; *0.17*)	0.24 (-0.33, 0.81; *0.41*)	0.41 (-0.11, 0.93; *0.12*)	-0.020 (-0.14, 0.10; *0.74*)	-0.050 (-0.29, 0.20; *0.70*)
**Model E**[a]					
Maternal Cd	-1.1 (-1.7, -0.46; *0.001*)	-0.61 (-1.3, 0.010; *0.066*)	-0.89 (-1.5, -0.29; *0.004*)	0.026 (-0.11, 0.17; *0.71*)	0.064 (-0.23, 0.35; *0.67*)
Maternal As	-0.55 (-0.93, -0.18; *0.004*)	-0.10 (-0.49, 0.28; *0.60*)	-0.40 (-0.76, -0.051; *0.025*)	0.031 (-0.053, 0.11; *0.47*)	0.037 (-0.13, 0.21; *0.67*)
Maternal Pb	0.45 (-0.29, 1.2; *0.23*)	-0.21 (-0.98, 0.56; *0.60*)	0.052 (-0.65, 0.76; *0.88*)	0.055 (-0.11, 0.22; *0.51*)	0.29 (-0.050, 0.63; *0.094*)
**Model F**[a]					
Concurrent Cd	-0.40 (-0.90, 0.098; *0.12*)	-0.74 (-1.3, -0.21; *0.006*)	-0.61 (-1.1, -0.14; *0.011*)	-0.057 (-0.17, 0.053; *0.31*)	0.11 (-0.12, 0.34; *0.34*)
Concurrent As	-0.47 (-0.81, -0.14; *0.005*)	0.12 (-0.23, 0.48; *0.50*)	-0.26 (-0.57, 0.062; *0.12*)	0.039 (-0.035, 0.11; *0.30*)	0.042 (-0.11, 0.19; *0.59*)
Concurrent Pb	0.41 (-0.13, 0.96; *0.14*)	0.24 (-0.34, 0.82; *0.41*)	0.44 (-0.084, 0.95; *0.10*)	-0.016 (-0.14, 0.11; *0.80*)	-0.039 (-0.29, 0.21; *0.76*)

[a]Besides the variables presented below each model we further adjusted all models for age at testing, tester, sex, birth order, birth weight, HAZ at 5 years, HOME, maternal BMI in early pregnancy, maternal IQ, and SES.
